# PAX5-Negative Classical Hodgkin Lymphoma: A Case Report of a Rare Entity and Review of the Literature

**DOI:** 10.1155/2017/7531729

**Published:** 2017-10-04

**Authors:** Elham Vali Betts, Denis M. Dwyre, Huan-You Wang, Hooman H. Rashidi

**Affiliations:** ^1^Department of Pathology and Laboratory Medicine, University of California, Davis, Davis, CA, USA; ^2^Department of Pathology, University of California, San Diego, La Jolla, CA, USA

## Abstract

Classical Hodgkin lymphoma (CHL) is recognized as a B-cell neoplasm arising from germinal center or postgerminal center B-cells. The hallmark of CHL is the presence of CD30 (+) Hodgkin and Reed-Sternberg (HRS) cells with dim expression of PAX5. Nearly all of the HRS cells are positive for PAX5. However, a small minority of HRS cells may lack PAX5 expression, which can cause a diagnostic dilemma. Herein we describe two cases of PAX5-negative CHL and review of the English literature on this very rare entity. It is crucial to be aware of this phenomenon, which in some cases may lead to misdiagnosis and may ultimately adversely affect patient's management.

## 1. Introduction

Classical Hodgkin lymphoma (CHL) per WHO 2008 is a clonal lymphoid neoplasm. CHL contains Reed-Sternberg (RS) cells in a background of a nonneoplastic inflammatory infiltrate including lymphocytes, eosinophils, neutrophils, histiocytes, and plasma cells [1]. Kanzler et al. microdisected and analyzed the RS cells from frozen tissue and showed nearly all RS cells carry immunoglobulin (Ig) heavy and light chain rearrangement, which supported the B-cell origin of these neoplasms [2]. Moreover, identical IgHV gene rearrangement was detected between RS cells of a given case, which showed the monoclonality of these cells [2]. RS cells show somatic hypermutation in the IgHV gene and since these mutations occur in the proliferating B-cells in germinal centers (GC), they are recognized to arise from GC or post-GC B-cells. The RS cells therefore are expected to express B-cell specific markers. According to WHO, the most specific B-cell marker is CD19 and since PAX5 is closely tied to this molecule, nearly all of the RS cells are reported to express PAX5 by immunohistochemistry. RS cells, however, are typically negative for CD19 [3]. The PAX5 acts as a transcriptional factor that is expressed by B-cells and its binding sites serve as promotors for certain B-cell-specific genes such as those that promote CD19 expression [4]. The expression of PAX5 is reduced and in rare cases of CHL, PAX5 expression is absent in RSC. This has been postulated to be caused by compromised B-cell specific transcription machinery and inactivity of immunoglobulin promoters, which results in low levels to absent expression of several B-cell-restricted transcription factors such as PAX5 and OCT2 [5]. Hence, the PAX5 negative cases of CHL are extremely rare and pose a major diagnostic challenge for hematopathologists. In review of the English literature, no known large case series of PAX5 negative CHL cases are noted and it is extremely important to have a review of the literature on this extremely rare entity.

## 2. Case Presentation

### 2.1. Case  1

**Figure 1:**
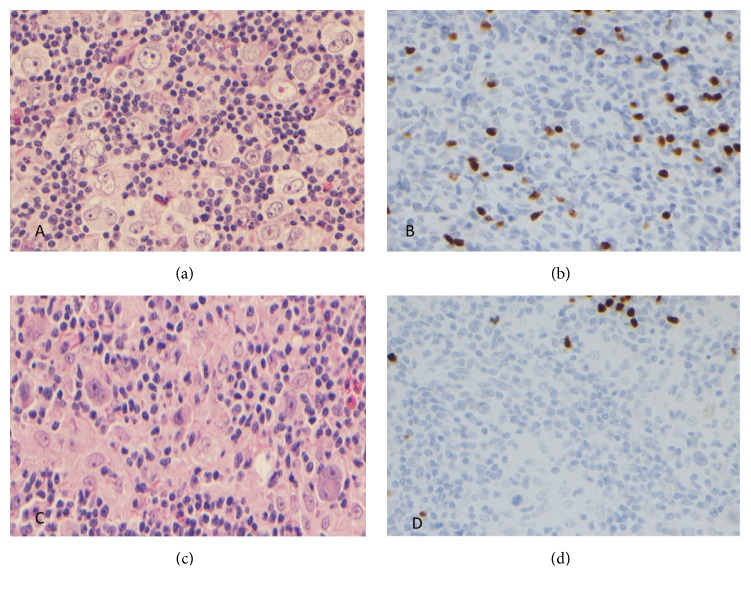
(a and c) Large atypical lymphoid cells with one to more nuclei, prominent nucleoli, and abundant cytoplasm consistent with RS cells and Hodgkin cells on the hematoxylin and eosin stain. (b and d) The large atypical cells are negative for PAX5 expression.

A 22-year-old male presented with a neck mass, night sweats, and weight loss. The excisional biopsy showed an effaced node involved by a nodular lymphohistiocytic infiltrate separated by fibrocollagenous bands. Among the background inflammatory cells were large atypical lymphoid cells with one to more nuclei, prominent nucleoli, and abundant pale cytoplasm, resembling Hodgkin Reed-Sternberg (HRS) cells (Figure 1(a)). These large atypical cells were positive for CD30 and focally positive for CD15. They were negative for PAX5 (Figure 1(b)), OCT2, BOB1, CD20, BCL6, CD2, CD3, CD4, CD5, CD7, CD8, and ALK1. T-cell gene rearrangement was negative which along with negative expression of pan-T-cell markers and ALK excludes anaplastic large cell lymphoma (ALCL). The H&E histology along with the immunohistochemical profile confirmed the suspected morphologic diagnosis of a CHL, nodular sclerosis subtype. Patient has been receiving treatment and has undergone allogeneic bone marrow transplant.

### 2.2. Case  2

A 53-year-old HIV-negative male presented with discomfort at the base of the neck, which was unresponsive to antibiotic therapy. A mass was noted, and a fine needle aspiration (FNA) was performed of the mass. The FNA showed scattered large atypical lymphoid cells. On a follow-up excisional biopsy, H&E sections of a lymph node showed an effaced nodal architecture with prominent nonnecrotizing granulomas and a mixed infiltrate of small lymphoid cells, histiocytes, and eosinophils with intermingled large atypical cells. The majority of the large cells were mononucleated cells with prominent large cherry red nucleoli and moderate to abundant eosinophilic cytoplasm, consistent with Hodgkin cells (Figure 1(c)). Occasional classical binucleated RS cells were also noted. These large cells were positive for CD30, CD15 (subset), CD79a (small subset), and MUM1 but were negative for CD20, CD45, OCT2, PAX5 (Figure 1(d)), CD2, CD3, CD4, CD5, CD7 CD8, granzyme-B, perforin, and ALK1. In this case expression of CD79a in a subset of RSC indicates the B-cell origin of the CD30 positive malignant cells. The T-cell gene rearrangement was negative which along with negative expression of the T-cell markers, ALK, and cytotoxic markers excludes ALCL. No acid fast organisms were noted by AFB stain. Subsequently, a diagnosis of a CHL, mixed cellularity subtype was rendered. Patient has undergone six cycles of chemotherapy and has been responding well to the treatment.

## 3. Discussion

PAX5 negative CHLs are extremely rare since CHL is believed to be a B-cell neoplasm. PAX5 is a nuclear transcription factor and, among the hematopoietic malignancies, the expression of this marker is mostly restricted to B-cells [6, 7]. The gene expression of PAX5 is increased during B-cell maturation and PAX5 expression has been shown to regulate B-cell proliferation and immunoglobulin secretion [6]. Hence, the absence of PAX5 in CHL is a very unusual finding and warrants further investigation. A study by Desouki et al. showed five of 39 cases of CHL were negative for PAX5 by immunohistochemical staining; of the five CHL cases noted in this study, two were mixed cellularity, two were nodular sclerosis, and one CHL was not otherwise specified [6]. Hertel et al. showed 4 cases of PAX5-negative classical Hodgkin lymphoma nodular sclerosis and one case of CHL, mixed cellularity type from 18 cases evaluated in the study [5]. In another study performed by Johri et al. one case out of 24 cases of CHL lacked expression of PAX5 [8]. A study by Foss et al. showed 3 cases of CHL without expression of PAX5 by immunohistochemistry out of 31 cases that were evaluated [9]. In a study performed by Nguyen et al., they showed two cases of PAX5 negative from 74 cases of CHL evaluated in their study [10] (Table 1).

In the differential diagnoses of PAX5 negative CHL, ALCL (a T-cell lymphoma) has to be considered and ruled out, typically by immunohistochemical staining and gene rearrangement studies. Unlike HRS cells in CHL, large cells in ALCL are commonly positive for CD45 and may express EMA [11]. In ALK positive ALCL, the tumor cells may have loss of many of the pan T-cell markers in addition to being positive for ALK1 staining. In the ALK negative ALCL cases, the tumor cells nearly always express CD2 and most are CD4 positive [11]. Cytotoxic markers, such as perforin and granzyme-B, are expressed in ALCL. Additionally, T-cell receptor gene rearrangement studies are helpful in such cases as molecular findings indicative of a clonally restricted T-cell population would strongly favor a form of T-non-Hodgkin lymphoma (T-NHL)/ALCL over CHL. ALCL should be negative for PAX5. However, rare cases of ALCL with expression of PAX5 have been reported [12, 13]. PAX5 expression in these cases has been reported to be due to extra copies of PAX5 and not PAX5 rearrangement [13]. It is important to be aware of both of these entities, PAX5-negative CHL and PAX5-positive ALCL, and use extensive immunohistochemical stains along with gene rearrangement studies to define the origin of the neoplastic cells.

The clinical significance of the lack of PAX5 staining in CHL is unknown. A very small study of PAX5-negative CHL cases suggested that patients with PAX5-negative CHL may have worse clinical outcomes, when compared to typical PAX5-positive CHL. These patients are more prone to relapse or short-ended progression free survival [11]. The study was very small and a larger evaluation of patients with this rare entity would need to be studied before making a definitive assessment of such potential prognostic significance. Additionally, the diagnosis of a PAX5-negative CHL should be done with extreme caution and only when other mimickers have been definitively ruled out.

## Figures and Tables

**Table 1:** PAX5-negative HRS cells in different variants of CHL.

Reported cases of PAX5-negative CHL	
	PAX5-negative CHL

Desouki et al.	
MCHL^1^	2
NSHL^2^	2
LRHL^3^	0
CHL, NOS^4^	1
Total	5
Hertel et al.	
NSHL	4
MCHL	1
Johri et al.	
CHL	1
Nguyen et al.	
CHL	2
Vali Betts et al.	
CHL, NS type^5^	1
CHL, MC type^6^	1

^1^Mixed cellularity CHL, ^2^nodular sclerosis CHL, ^3^lymphocyte rich CHL, ^4^CHL, not otherwise specified, ^5^CHL, nodular sclerosis type, and ^6^CHL, mixed cellularity type.
